# Insulin Treatment Forces Arteriogenesis in Diabetes Mellitus by Upregulation of the Early Growth Response-1 (Egr-1) Pathway in Mice

**DOI:** 10.3390/ijms20133320

**Published:** 2019-07-05

**Authors:** Senthilkumar Thulasingam, Sundar Krishnasamy, David Raj C., Manuel Lasch, Srinivasan Vedantham, Elisabeth Deindl

**Affiliations:** 1 School of Chemical and Biotechnology, SASTRA Deemed to be University, Thanjavur 613401, India; 2 Department of Otorhinolaryngology, Head & Neck Surgery, University Hospital, LMU Munich, 81377 Munich, Germany; 3 Walter-Brendel-Centre of Experimental Medicine, University Hospital, LMU Munich, 81377 Munich, Germany

**Keywords:** arteriogenesis, endothelial cells, smooth muscle cells, diabetes mellitus, Egr-1, streptozotocin, collateral arteries, insulin

## Abstract

The process of arteriogenesis is severely compromised in patients with diabetes mellitus (DM). Earlier studies have reported the importance of *Egr-1* in promoting collateral outward remodeling. However, the role of *Egr-1* in the presence of DM in outward vessel remodeling was not studied. We hypothesized that *Egr-1* expression may be compromised in DM which may lead to impaired collateral vessel growth. Here, we investigated the relevance of the transcription factor *Egr-1* for the process of collateral artery growth in diabetic mice. Induction of arteriogenesis by femoral artery ligation resulted in an increased expression of Egr-1 on mRNA and protein level but was severely compromised in streptozotocin-induced diabetic mice. Diabetes mellitus mice showed a significantly reduced expression of *Egr-1* endothelial downstream genes Intercellular Adhesion Molecule-1 (*ICAM-1*) and urokinase Plasminogen Activator (*uPA*), relevant for extravasation of leukocytes which promote arteriogenesis. Fluorescent-activated cell sorting analyses confirmed reduced leukocyte recruitment. Diabetes mellitus mice showed a reduced expression of the proliferation marker Ki-67 in growing collaterals whose luminal diameters were also reduced. The Splicing Factor-1 (SF-1), which is critical for smooth muscle cell proliferation and phenotype switch, was found to be elevated in collaterals of DM mice. Treatment of DM mice with insulin normalized the expression of *Egr-1* and its downstream targets and restored leukocyte recruitment. SF-1 expression and the diameter of growing collaterals were normalized by insulin treatment as well. In summary, our results showed that Egr-1 signaling was impaired in DM mice; however, it can be rescued by insulin treatment.

## 1. Introduction

Peripheral artery disease (PAD) is one of the common vascular complications in diabetes mellitus [1]. Patients with PAD exhibit poor lower extremity function and develop critical limb ischemia and ulceration, ultimately leading to limb amputation [2,3,4]. Moreover, compared to healthy individuals, diabetic patients with PAD exhibit cardiovascular co-morbidities, neuropathy, and higher mortality [5,6,7,8]. Patients with PAD show poorer outcomes after leg bypass surgery with higher incidence of restenosis, longer hospitalization, and reduced amputation-free survival [8,9,10,11].

Arteriogenesis, which is an endothelial dependent process [12], is characterized by outward remodeling of pre-existing anastomoses in conducting arteries, and through this process, the blood flow to peripheral tissues can largely be restored. The process of collateral artery growth is strongly dependent on perivascular recruitment and accumulation of leukocytes, particularly macrophages, which supply growth factors and cytokines to the growing vessel [13]. In addition, lymphocytes accumulate in the perivascular space [14]; however, little is known about their function. It is well established that the presence of DM limits the process of arteriogenesis [15]. Indeed, it has been previously shown by van Weel et al. [16] that reperfusion recovery after femoral artery ligation (FAL) was significantly reduced in streptozotocin (STZ)-induced diabetic mice, as shown by laser Doppler perfusion measurements. The exact mechanisms through which impairment of arteriogenesis in DM occurs are not clear. Elevated vasomotor function attenuating the sensing of shear stress and defects in downstream monocyte signaling are reported as major contributors to the vascular impairments seen in arteriogenesis [17].

Early growth response-1 (*Egr-1*) is a zinc finger transcription factor, which is expressed after exposure of cells to mediators associated with growth and differentiation [18]. Several studies have shown a link between the activation of *Egr-1* through hypoxia, ischemia/reperfusion, mechanical stress, shear stress, emphysema, atherosclerosis, and acute vascular injury [19]. Early growth response-1 has been reported to play a critical role in a hind limb ischemia model [20], and in a separate study, it was shown that adenoviral-mediated *Egr-1* delivery improved perfusion recovery [21]. Early growth response-1 is important for leukocyte recruitment and vascular cell proliferation during arteriogenesis in vivo in mice subjected to FAL [22]. Thus, with increased *Egr-1* expression playing a critical role in both arteriogenesis and regulation under hyperglycemic conditions, the present study was performed to understand the influence of DM on *Egr-1* expression and its consequent biological events. We hypothesized that *Egr-1* expression may be compromised in DM which may lead to impaired collateral vessel growth. In the present study, we investigated the role of *Egr-1* in collateral artery growth in vivo in streptozotocin-induced diabetic mice employing a hind limb model in which arteriogenesis was induced by FAL.

## 2. Results

C57Bl6J mice were made diabetic using streptozotocin. The diabetic mice showed a significant rise in blood glucose levels (328 ± 38 mg/dL) compared to non-diabetic mice (144 ± 10 mg/dl) 24 h post-ligation. In accordance with this, diabetic mice treated with insulin showed a significant decrease in glucose levels (216 ± 35 mg/dL) compared to the diabetic group (Table 1) at the same timepoint.

### 2.1. Reduced Upregulation of Egr-1 in Growing Collaterals of Diabetic Mice

**Figure 1:**
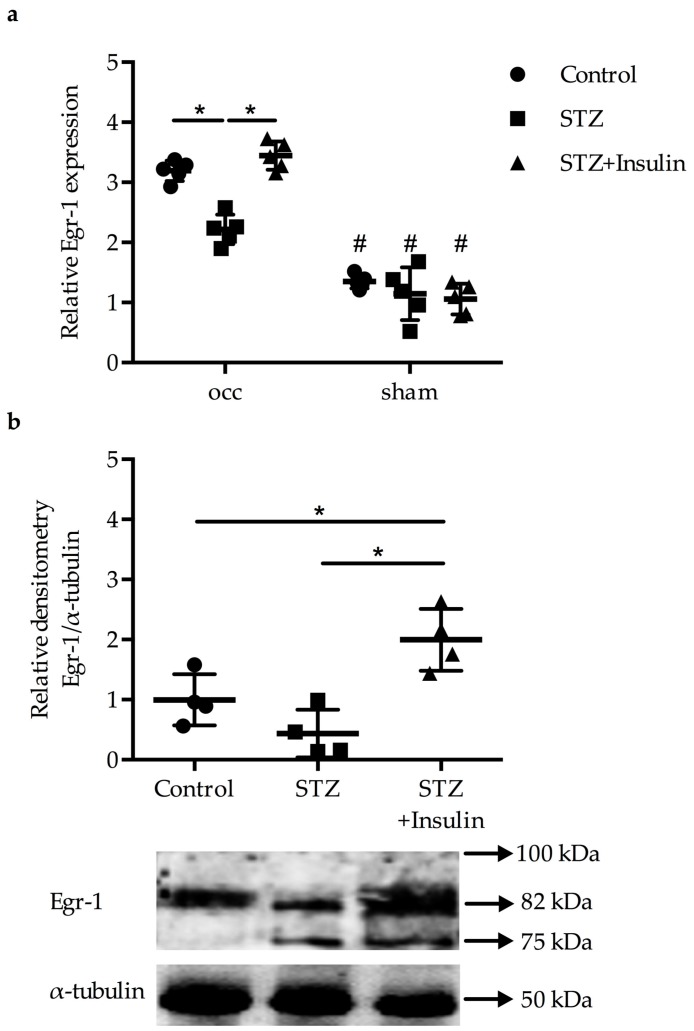
Gene expression studies of (**a**) mRNA levels and (**b**) protein levels of collateral arteries obtained from occluded and sham-operated mice 24 h after the surgical procedure. (**a**) Dot plots representing the results of qRT-PCR analyses (*n* = 5 per group, * *p* < 0.05 (each group compared to each other group), ^#^ *p* < 0.05 compared to corresponding occ group from one-way ANOVA with Bonferroni’s multiple comparison test). Results were normalized to the expression level of the 18S rRNA. (**b**) Quantitative analyses (upper panel) and corresponding representative pictures of a Western blot (lower panel) showing the protein expression of Egr-1 as well as of α-tubulin, which was used for normalization, 24 h post-FAL (*n* = 4 per group, * *p* < 0.05 (each group compared to each other group) from one-way ANOVA with Bonferroni’s multiple comparison test).

The Egr-1 mRNA expression levels were significantly increased in growing collaterals of control mice, diabetic mice, and diabetic mice treated with insulin compared to resting collaterals isolated from the sham-operated side (Figure 1a). However, the increase in the expression levels of *Egr-1* was significantly less pronounced in diabetic mice compared to non-diabetic mice and control mice 24 h post-FAL (2**^^−ddCT^**, 3.19 ± 0.172 versus 22 ± 0.25, *p* < 0.05) (Figure 1a). Treatment of diabetic mice with insulin significantly increased *Egr-1* expression compared to the diabetic mice (3.45 ± 0.25) (Figure 1a), resulting in similar levels as in the control group. Western blot analyses revealed a significantly lower expression of Egr-1 protein in the diabetic group compared to both the non-diabetic control group and the insulin treated group (Figure 1b) 24 h after the surgical procedure.

### 2.2. Expression of Egr-1 Downstream Genes in Collaterals of Diabetic Mice Was Restored by Insulin Treatment

**Figure 2:**
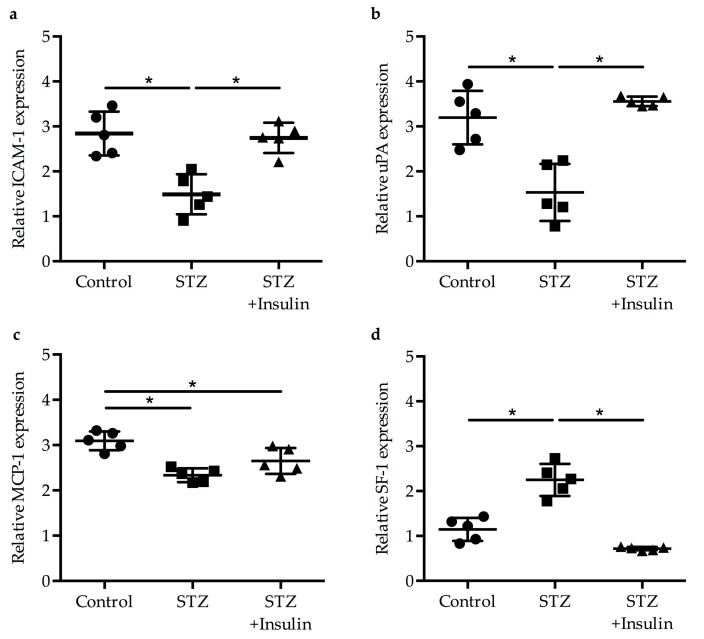
Gene expression of downstream targets of *Egr-1* in collateral tissues obtained from mice after induction of arteriogenesis was performed by qRT-PCR (*n* = 5 in each group, * *p* < 0.05 (each group compared to each other group) using a one-way ANOVA with Bonferroni’s multiple comparison test). The relative expression is represented as 2**^^−ddCT^**. The relative expressions of (**a**) *ICAM-1*, (**b***) uPA,* (**c**) *MCP-1*, and (**d**) SF-1 were normalized to the expression level of the 18S rRNA.

The mRNA expression levels of Egr-1 downstream target genes, namely, Intercellular Adhesion Molecule-1 (ICAM-1), urokinase Plasminogen Activator (uPA), and Monocyte Chemoattractant Protein-1 (MCP-1) [18,23,24], were measured via qRT-PCR. Our results revealed decreased expression of ICAM-1 (Figure 2a) and uPA (Figure 2b) in the diabetic group compared to the non-diabetic control group (1.49 ± 0.49 versus 2.84 ± 0.49 and 1.53 ± 0.64 versus 3.2 ± 0.60, respectively). Mice treated with insulin showed a significant rise in ICAM-1 (2.35 ± 0.34) and uPA (3.56 ± 0.10) expression compared to the diabetic group. The expression of *MCP-1* decreased in diabetic mice (2.34 ± 0.15); however, its expression levels did not significantly rise upon treatment with insulin (2.65 ± 0.29) (Figure 2c). As previous results have shown that the transcriptional repressor Splicing Factor-1 (SF-1), which controls smooth muscle cell proliferation, is upregulated in growing collaterals during the process of arteriogenesis [22], we also investigated the expression level of the corresponding transcript. Our data showed a significant upregulation of SF-1 in the diabetic group (2.25 ± 0.36) compared to the non-diabetic group (1.15 ± 0.26), but its expression level was normalized again by insulin treatment (0.72 ± 0.04) (Figure 2d).

### 2.3. Insulin Treatment Restored Vessel Growth in Diabetic Mice

**Figure 3:**
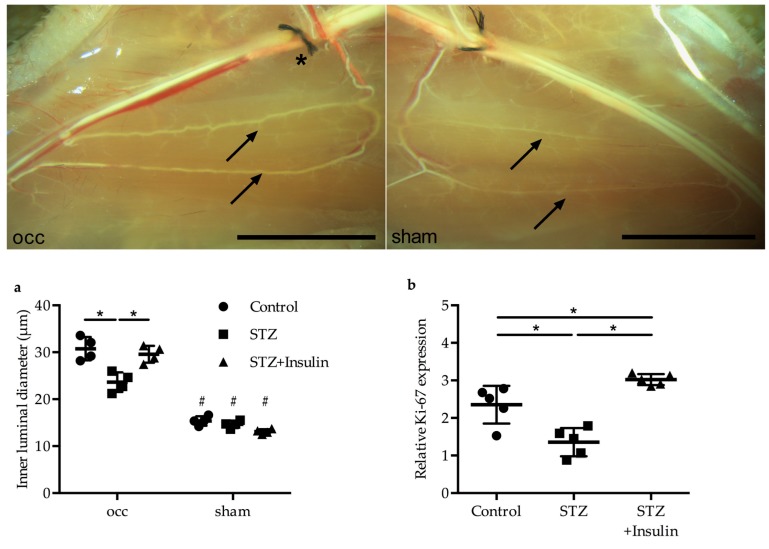
Upper panel: pictures of superficial collateral arteries of STZ-treated mice 7 days after femoral artery ligation (FAL, occ, left picture) or sham operation (right picture). Arrows indicate pre-existing (resting) collaterals of the sham-operated site or growth-induced collaterals of the experimental site. The ligation (*) of the femoral artery was executed downstream of the profound artery. Scale bars: 5 mm. Lower panel: (**a**) dot plots represent the inner luminal vessel diameter measured 7 days post-FAL (*n* = 4 per group, at least two collateral arteries per mouse and two sections were evaluated, * *p* < 0.05 (each group compared to each other group), ^#^ *p* < 0.05 compared to the corresponding occ group using a one-way ANOVA with Bonferroni’s multiple comparison test). (**b**) Dot plots show the expression level of the proliferation marker Ki-67 in collaterals of control, STZ, and STZ + insulin-treated mice 24 h after FAL. Results were normalized to the expression level of the 18S rRNA (*n* = 5 per group, * *p* < 0.05 (each group compared to each other group) using a one-way ANOVA with Bonferroni’s multiple comparison test).

The luminal collateral vessel diameter, which was measured 7 days post-FAL, was found to be significantly decreased in the diabetic group (23.61 ± 2.1 µm) compared to the control group (30.76 ± 2.5 µm); however, it was restored to control levels by insulin treatment (29.58 ± 1.8 µm) (Figure 3a). Moreover, qRT-PCR results on the cell proliferation marker Ki-67 revealed significantly reduced Ki-67 mRNA expression in the diabetic group (1.36 ± 0.37) compared to the non-diabetic control group (2.35 ± 0.50); however, Ki-67 expression levels increased when STZ mice were treated with insulin (3.02 ± 0.15) (Figure 3b).

### 2.4. Diminished Leukocyte Infiltration Was Improved in Diabetic Mice by Insulin Treatment

**Figure 4:**
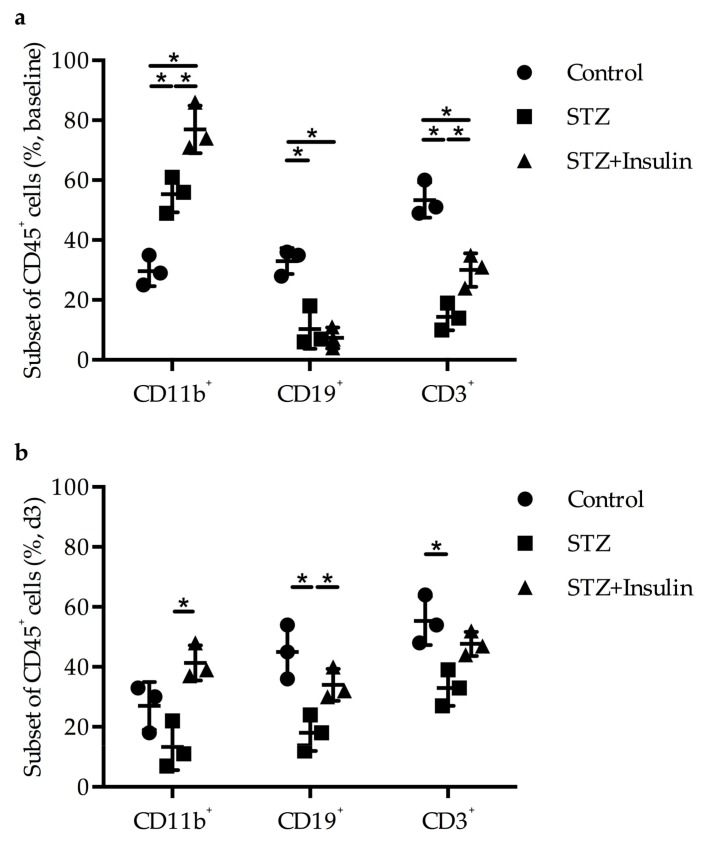
Dot plots represent the results of FACS analyses on CD11b^+^, CD19^+^, and CD3^+^ cells performed on (**a**) whole blood of control, STZ, and STZ + insulin-treated mice without any surgical treatment and on (**b**) adductor muscles isolated from mice 3 days post-FAL (**a**,**b**: *n* = 3 per group, each group compared to each other group using a two-way ANOVA with Bonferroni’s multiple comparison test).

Fluorescent-activated cell sorting (FACS) studies from blood collected from non-ligated mice showed significantly increased levels of CD11b^+^ cells in diabetic (55%) and insulin-treated groups (77%) compared to the non-diabetic control group (33%) with respect to CD45^+^ cells, whereas the levels of CD19^+^ or CD3^+^ cells decreased in both the diabetic (10% and 14%, respectively) and the insulin-treated group (7% and 30%, respectively) (Figure 4a). There were no significant changes in leukocyte count in the adductor muscle of sham-operated mice in STZ-treated and STZ and insulin-treated mice compared to control mice (data not shown). However, in diabetic mice, there was a significant decrease in CD11b^+^ (27% versus 13%), CD19^+^ (45% versus 18%), and CD3^+^ (55% versus 33%) cells three days post-ligation compared to control mice. Interestingly, insulin treatment increased the number of leukocytes in STZ-induced DM mice (Figure 4b).

## 3. Discussion

**Figure 5:**
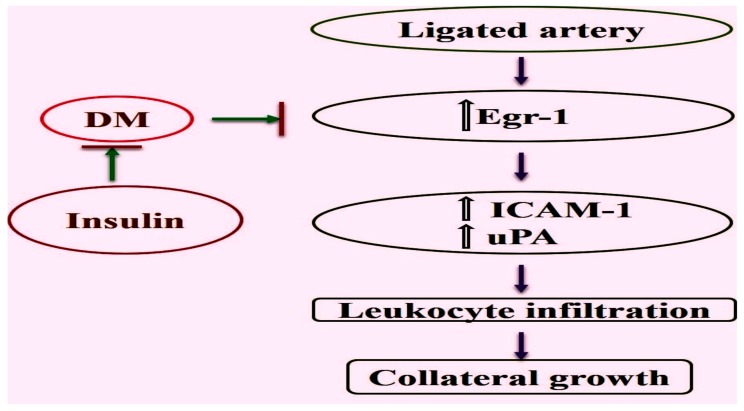
Graphical representation showing the mechanisms by which Egr-1 controls leukocyte recruitment, and hence, the process of collateral artery growth. While this process is impaired in DM due to the reduced *Egr-1* expression, it can be rescued by insulin treatment.

PAD is one of the common complications of DM that inflicts substantial damage to the lower limbs and has a very poor outcome. In mice, it was shown that impaired arteriogenesis is a major problem in hypercholesterolemia and DM [16]. While in the study by van Weel et al. [16], the influence of DM on perfusion recovery was less compared to that of hypercholesterolemia, our study showed a more pronounced impact of ligation on collateral vessel growth in DM. This may be due to the different experimental setup, age groups of animals under study, timepoints measured, and animal strains used. The mechanism by which impaired arteriogenesis occurs in DM is not fully understood. Earlier, Pagel et al. [22] reported the importance of *Egr-1* in promoting collateral outward remodeling through augmenting leukocyte infiltration and endothelial cell proliferation. In the present study, we report that there was a decreased expression of Egr-1 at the transcript and protein levels in collaterals of mice rendered diabetic by administration of streptozotocin. The decreased expression of *Egr-1* correlated with decreased collateral artery diameter in the diabetic mice. Important downstream targets of *Egr-1*, namely, *ICAM-1* and *uPA*, were found to be decreased, too. *ICAM-1* plays a critical role in endothelial monocyte adhesion, which is essential for arteriogenesis. An earlier study identified an upregulation of ICAM-1 mRNA in growing collateral arteries after induction of arteriogenesis by femoral artery ligation [25]. Moreover, it was shown that the process of arteriogenesis is reduced in ICAM-1 deficient mice [26]. Treatment of the diabetic mice with insulin improved the expression of *Egr-1*, increased the collateral artery diameter, and normalized the expression of downstream targets of *Egr-1*. A proposed model is shown in Figure 5.

Earlier studies have clearly shown that induction of arteriogenesis in C57Bl6 mice by FAL resulted in an increased expression of *Egr-1* at the transcript and the protein levels in growing collaterals [22]. Interestingly, *Egr-1* deficient mice have been shown to have increased basal levels of CD11b^+^ monocytes in the peripheral blood; however, levels of collateral perivascular macrophages as well as CD3^+^ T cells and CD19^+^ B cells in adductor muscles harvesting growing collaterals were reduced. Moreover, FAL in Egr-1−/− mice was associated with poor leukocyte recruitment and reduced collateral artery growth [22]. Our results showed that induction of DM, which was associated with reduced expression levels of *Egr-1*, also resulted in increased systemic levels of CD11b^+^ cells, and after induction of arteriogenesis, in reduced levels of CD11b^+^, CD3^+^, and CD19^+^ cells. Interestingly enough, treatment with insulin rescued perivascular leukocyte counts.

Decreased expression of *Egr-1* in DM mice in our study may support earlier findings showing that *Egr-1* is critical for collateral vessel development and that functional regulation of *Egr-1* may be compromised in DM. Evidence of induction and expression of *Egr-1* by elevated levels of glucose in murine glomerular endothelial cells and aortic smooth muscle cells have been reported. Exposure to insulin or high concentrations of D-glucose increased the expression of Egr-1 on the mRNA and protein level in glomerular endothelial cells and increased its promoter activity irrespectively of the concentration of insulin [27,28]. *TNF-α* is downstream of *Egr-1* and induces the expression of *MCP-1*. However, in arteriogenesis, increased levels of TNF-α, relevant for *MCP-1* expression, are dependent on mast cell activation [14]. Vedantham et. al. demonstrated a novel mechanism linking glucose metabolism to increased inflammatory and prothrombotic signaling in diabetic atherosclerosis via activation and post-translational modification of *Egr-1*. Hyperglycemia-induced hyper-acetylation of Egr-1 in endothelial cells was reported to be an important event linking diabetes to accelerated atherosclerosis [29]. Though acetylation of Egr-1 was not studied, it will be interesting to pursue future studies to understand the role of Egr-1 in the pathophysiology of arteriogenesis. Our observations in this study are contrary to those observed in diabetic atherosclerosis. There may be a possibility of additional regulation of Egr-1, as indicated by the shift in bands of Egr-1. Post-translational modifications of Egr-1 such as acetylation and phosphorylation have been reported to play an important role in the transcriptional activity and stability of Egr-1 [17,30]. Phosphorylation/dephosphorylation events may act as regulators for restricting the function of Egr-1. Furthermore, *SP-1* has been reported to compete for DNA binding sites of *Egr-1* [17]. Recently, a splice form of *Egr-1* was reported which lacks the N-terminal activation domain between amino acids 141 and 278 [31]. It will be interesting to further understand the mechanism through which hyperglycemia interferes with *Egr-1* upregulation during the process of arteriogenesis.

Leukocyte infiltration mediated through downstream target genes of *Egr-1*, namely, *ICAM-1, uPA* and *MCP-1*, was found to be decreased during collateral artery growth in diabetic mice. Endothelial uPA is vital for neutrophil adherence to the endothelial cells [32]. Neutrophils accumulate around day 1 after FAL in the perivascular space of growing collaterals and have a relevant function in the recruitment of macrophages and lymphocytes, which appear at day 3 [14]. Indeed, it has been shown that *uPA* deficiency is associated with reduced perivascular leukocyte accumulation and results in reduced collateral artery growth after induction of arteriogenesis via FAL [33]. These data comply with reported findings that *Egr-1* mediates leukocyte infiltration through activation of the abovementioned genes in arteriogenesis [21]. Furthermore, the expression of the cell proliferation marker Ki-67 was found to be decreased in the growing collaterals of diabetic mice, highlighting the fact that, indeed, there was a decrease in the proliferation of vascular cells. SF-1, an important transcriptional repressor critical for smooth muscle proliferation and phenotype switch [33], was found to be elevated in our study in support of an earlier report demonstrating that Egr-1−/− mice exhibited increased expression of SF-1 in growing collateral arteries [21]. One of the interesting findings of our study as the beneficial effect of insulin on collateral artery growth in DM mice. SF-1 regulates gene expression of pro-inflammatory cytokines in smooth muscle cells [33] and antagonizes platelet-derived growth factor BB (PDGF-BB)-induced growth and differentiation of vascular cells [34]. Several earlier reports have shown a regulation of *Egr-1* by insulin. Furthermore, Gousseva et al. [28] have reported an insulin-mediated increase in *Egr-1* promoter activity and cell proliferation in bovine aortic smooth muscle cells. Egr-1 has a role in adipocyte insulin resistance through activation of the MAPK-ERK pathway [35]. Our studies clearly show that diabetic mice treated with insulin show an increased expression of *Egr-1*, accompanied by an augmented expression of *Egr-1* downstream target genes relevant for leukocyte recruitment. Indeed, our results show that insulin treatment, moreover, goes along with increased numbers of leukocytes—relevant for the process of arteriogenesis—in collateral harboring muscles.

In summary, *Egr-1* expression decreased after induction of arteriogenesis in growing collaterals of streptozotocin-induced diabetic mice compared to control mice. Decreased *Egr-1* expression led to poor collateral growth. Insulin treatment, however, normalized the *Egr-1* expression, thereby promoting arteriogenesis. Though this was an observational study, this study assumes significance as it is the first time associating *Egr-1* with impaired arteriogenesis in DM. It will be interesting to see whether the same processes occur in patients with DM. Further investigations exploring the mechanism by which hyperglycemia suppresses *Egr-1* expression during the process of arteriogenesis will lead to better understanding of impaired arteriogenesis in DM.

## 4. Materials and Methods

### 4.1. Animal Studies

All studies with mice were performed after approval by the Institutional and Local Animal Ethics Committee (CPCSEA Approval number: H01/SASTRA/IAEC/RPP-23/12/15). Male C57B6NTac (Taconic Biosciences, USA) mice were procured through Vivo Biotech Ltd., Telangana, India and were maintained in an air-conditioned room (25 °C) with a 12 h light/12 h dark cycle. Feed and water were provided ad libitum to all the animals. Mice were rendered diabetic by treating them with STZ according to published protocols [36]. Briefly, eight-week-old male mice were made diabetic by administration of 50 mg/kg STZ dissolved in fresh citrate buffer (0.05 mol/L, pH 4.5) i.p. per day for five consecutive days. Those mice displaying blood glucose levels ≥ 250 mg/dL were considered diabetic (DM mice). The non-diabetic (NDM mice) control mice received citrate buffer alone. One group of mice received insulin after confirmation of hyperglycemia. Insulin was administered subcutaneously at a daily dose of 0.20 mL/100 g (4–5 U) until the end of the experiment.

### 4.2. Femoral Artery Ligation and Collection of Collaterals

Femoral artery ligation was performed as published earlier [37]. In brief, using a silk braided suture (0/7) the right femoral artery was ligated distally from the origin of the profunda femoris branching, while the left leg was sham operated. Adductor muscles and collateral arteries were collected as previously described [20]. To carry out fluorescent-activated cell sorting (FACS) analyses, adductor muscles were collected 3 days after the surgical procedure and for histological analysis, the adductor muscles were harvested 7 days after FAL. Gene expression studies on RNA and protein levels were performed with collateral arteries isolated 24 h after induction of arteriogenesis.

### 4.3. RNA Isolation and Quantitative Real-Time PCR Studies

Gene expression studies were performed using quantitative real time PCR (qRT-PCR). Total RNA was isolated using the RNeasy kit (TaKaRA). After DNase I (Qiagen, Hilden, Germany) digestion, one microgram of total RNA was reverse transcribed by random hexamers and Superscript RT-PCR System (Invitrogen, Carlsbad, CA, USA). After purification, the cDNA was used for qRT-PCR using specific primers for Egr-1, MCP-1, ICAM-1, SF-1, and Ki-67 [21]. Results were normalized to the expression level of the 18S rRNA.

### 4.4. FACS Analyses of Blood and Muscle Tissue

Whole blood withdrawn from the left ventricle 3 days post-ligation was analyzed by flow cytometry analyses (BD FACS Aria III, CA, USA) according to standard protocols. Furthermore, the adductor muscles from C57B6NTac mice were perfused with PBS (phosphate buffered saline) to eliminate the blood, harvested, and placed in small cell culture dishes. The tissue was cut into small pieces and digested 45 min at 37 °C using PBS buffer (50 mL) containing collagenase II (1 mg/mL), hyaluronidase (0.5 mg/mL) (both Sigma, St. Louis, MO, USA), dispase (1 mg/mL) (Gibco, Invitrogen, Carlsbad, CA, USA), and BSA (bovine serum albumin) (0.6 mg/mL) (Sigma). The suspension was then filtered with PBS/2%BSA through a 70 μm cell strainer (BD Falcon™), spun 10 min at 95 g, and the pellet finally resuspended in 100 μL PBS/2%BSA. The resulting cell suspension was analyzed by FACS using a panel of monoclonal antibodies against CD3 (T cells); (BioLegend Cat. No. 100201), CD11b (neutrophils, monocytes) (BioLegend Cat. No. 305902), CD19 (B cells) (BioLegend Cat. No. 115501), and CD45 (pan-leukocytes marker) (BioLegend Cat. No. 103101). Leukocyte populations were identified by fluorescence and scatter light characteristics. Cells from both peripheral blood and tissue were gated based on forward scatter (FSC-A)/side scatter (SSC-A). Leukocytes were identified by their positive staining with CD45. The final gating was based on CD45+/CD11b+, CD45+/CD19+, and CD45+/CD3+ cells (14).

### 4.5. Histological Analyses

Histological analyses were performed on adductor muscles (harboring collateral arteries) isolated from C57B6NTac mice as described earlier [20]. Briefly, 7 days after the surgical procedure both hind limbs were perfused with PBS containing 0.1% adenosine and 0.05% BSA (Sigma), then 4 min with fixing solution (4% buffered paraformaldehyde) via cannulation of the aorta. Thereafter, tissue samples were paraffin-embedded, cut in cross-sections, and H&E staining was performed to measure luminal collateral artery diameters.

### 4.6. Western Blot

Western blot analysis was performed on protein extracts, which were isolated from collaterals 24 h after femoral artery ligation or sham operation of DM and NDM mice according to standard procedures. Briefly, 30 µg of protein from all the tissue lysates was loaded onto a 10% sodium dodecyl sulfate (SDS) gel and ran at a power of 110 V. The protein in the gel was shifted to an immune-blot polyvinylidene difluoride (PVDF) membrane (1620112, Bio-Rad, USA) at 100 V for 1 h using Trans-Blot Turbo Transfer System (Bio-Rad). The blots were probed for Egr-1 protein (Egr-1 antibody (588)-Santa Cruz BioTechnology Cat.no #sc110). α-Tubulin served as a housekeeping protein (alpha tubulin antibody (B-7): Santa Cruz Biotechnology Cat.no. #sc-5286). The density of Egr-1 to α-tubulin was measured through the Quant-One software (Bio-Rad). The immunoblots are a representation of at least three independent experiments.

### 4.7. Statistical Analysis

All values are expressed as means ± SD unless mentioned. Statistical analyses were conducted as indicated in the figure legends using GraphPad software PRISM6 (GraphPad Software, USA). The limit of statistical significance was set at *p* < 0.05.

## Figures and Tables

**Table 1:** Plasma glucose levels measured 24 h post-Femoral Artery Ligation prior to sacrifice (*n* = 7 in each group).

Group	Random Plasma Glucose (mg/dL)
Control	144 ± 10
Streptozotocin	328 ± 38 *
Streptozotocin + Insulin	216 ± 35 **

* *p* < 0.05 compared to Control group, ** *p* < 0.05 compared to Streptozotocin group.

## Abbreviations

SF-1	Splicing Factor 1
DM	Diabetes Mellitus
NDM	Non-Diabetes Mellitus
Egr-1	Early growth response-1
uPA	urokinase Plasminogen Activator
MCP-1	Monocyte Chemoattractant Protein-1
ICAM-1	Intercellular Adhesion Molecule-1
FAL	Femoral Artery Ligation
STZ	Streptozotocin
PAD	Peripheral Artery Disease
